# The effects of safinamide on dysphagia in Parkinson’s disease

**DOI:** 10.1371/journal.pone.0286066

**Published:** 2023-05-25

**Authors:** Makito Hirano, Makoto Samukawa, Chiharu Isono, Yoshitaka Nagai

**Affiliations:** 1 Department of Neurology, Kindai University Faculty of Medicine, Ohnohigashi, Osakasayama, Osaka, Japan; 2 Department of Rehabilitation Medicine, Kindai University Faculty of Medicine, Ohnohigashi, Osakasayama, Osaka, Japan; Dokkyo Medical University: Dokkyo Ika Daigaku, JAPAN

## Abstract

Dysphagia is a potentially fatal symptom of Parkinson’s disease (PD) and is characterized by frequent silent aspiration, a risk factor for aspiration pneumonia. The transdermal dopamine agonist rotigotine alleviates dysphagia in patients with PD and is more effective than oral levodopa, suggesting the importance of continuous dopaminergic stimulation (CDS) in swallowing. Safinamide is a monoamine oxidase B (MAOB) inhibitor that facilitates CDS. In this retrospective open-label evaluator-blinded research, swallowing functions in nine patients with PD were examined using a video fluoroscopic swallowing study (VFSS) before and after treatment with 50 mg of oral safinamide. The VFSS results showed that safinamide significantly improved some swallowing measures during oral and pharyngeal phases, including oral transit time and pharyngeal transit time, without worsening of any measures. Notably, improvements in lip closure, an oral phase component, seemed to be most attributable to improvements in oral phase scores. In conclusion, a medicine for CDS may effectively improve swallowing functions in patients with PD. This is the first study to show that the MAOB inhibitor safinamide partly but significantly improves swallowing function in patients with PD.

## Introduction

Parkinson’s disease (PD) is one of the most common neurodegenerative diseases. It is characterized by motor symptoms, such as rhythmic shaking of the limbs (tremor), increased muscle tone (rigidity), reduced spontaneous movement (akinesia), and unsteady gait. In addition, patients with PD have nonmotor symptoms, including autonomic disturbances. The pathological hallmark of PD is the degeneration of dopaminergic neurons.

Abnormal swallowing, or dysphagia, is also commonly observed in PD and is a potentially fatal symptom with frequent silent aspiration, a risk factor for aspiration pneumonia [1–3]. Swallowing functions during oral and pharyngeal phases are especially important for such serious complications [4]. The oral phase is characterized mainly by voluntary movements, whereas the pharyngeal phase is associated mainly with the sequential reflexes of striatal muscles triggered by pharyngeal sensory inputs [4, 5]. PD affects voluntary movements as well as pharyngeal sensation, leading to dysphagia [4, 5].

The most commonly used medicine for PD is levodopa, which crosses the blood–brain barrier and is converted into dopamine in the neurons. This medicine may lead to improvements in swallowing efficiency; however, some controversial results have been reported [6, 7]. Dopamine agonists have also been used for the treatment of PD. Dopamine agonists directly stimulate dopamine receptors in the neurons. Among dopamine agonists, apomorphine and rotigotine have been reported to alleviate dysphagia in patients with PD [4, 8–10] and improve involuntary pharyngeal functions. Notably, a dosage of 2 mg/day of transdermal rotigotine (levodopa equivalent dose [LED], 60 mg/day) is more effective than a dosage of 200 mg/day of oral levodopa for dysphagia, suggesting that transdermal administration associated with continuous dopaminergic stimulation (CDS) is important in swallowing [9]. Despite their effectiveness, dopamine agonists have rare but serious adverse effects, such as the sudden onset of sleep, which precludes patients from driving [11]. Thus, medicines that result in CDS with few adverse events are advisable. Although our previous study including nine drug-naïve patients suggested that the monoamine oxidase B (MAOB) inhibitor rasagiline, which is capable of CDS, is effective for mild dysphagia in PD [12], investigations including mid- to late-stage PD patients are needed to reach to a conclusion.

Safinamide, another MAOB inhibitor, is used only as an add-on therapy to levodopa in Japan [13], and facilitates CDS [14]. We hypothesized that MAOB inhibition by safinamide would be effective in swallowing functions in mid- to late-stage PD. In this retrospective, open-label, evaluator-blinded study, the effects of safinamide on swallowing were examined using a video fluoroscopic swallowing study (VFSS) in nine patients with PD.

## Patients and methods

### Patients

A retrospective examination was conducted on nine consecutive Japanese patients with idiopathic PD (three men and six women; mean age ± SD, 78 ± 6 years) who received 50 mg/day oral safinamide (once a day) and underwent VFSS before and after treatment. The mean age of patients at the onset of PD was 72 ± 6 years, and the mean duration of the disease was 6 ± 3 years. All patients had been treated with other medicines for PD (387 ± 203 mg/day on LED) for 3 ± 3 years before safinamide administration. No PD medications other than safinamide were changed during the study. Only two of the nine patients received rotigotine. All patients were diagnosed with PD by board-certified neurologists in accordance with the UK Parkinson’s disease Society Brain Bank Clinical Diagnostic Criteria and had a Hoehn–Yahr grade of II to III. Only three of the nine patients had an apparent wearing-off phenomenon. Safinamide was prescribed due to worsening of the wearing-off phenomenon and parkinsonism. No apparent dementia, signs of upper motor neuron disease, painful or debilitating disorders, and previous history of stroke were noted among the participants. Cognitive tests were not performed because patients did not report cognitive decline. The enrolled patients did not receive specific training or rehabilitation of swallowing during safinamide treatment but were instructed by a neurologist to avoid the intake of anything difficult to swallow, keep appropriate positions of the head and trunk, and use a commercial xanthan gum-based thickener when necessary. This study was approved by our institutional review board. All participants provided written informed consent to publish their clinical data.

### VFSS

All patients were evaluated before and 32 ± 5 days (mean ± SD) after safinamide treatment. VFSS was performed during on-time in accordance with a previous method with a slight modification. The jelly mixed with the liquid barium was changed to a thickened barium solution, with jelly-solidified barium occasionally used at the request of the physician [15, 16]. The subject swallowed a diluted solution of barium (5 mL, 60% w/v) three times. If the swallowing problem was not severe, as indicated by the procedure and rating scales described below, then a concentrated solution of barium (150% w/v) was swallowed three times. In this step, the amount of barium solution was not restricted, and the subject was requested to swallow as usual. The consistency of the diluted and concentrated solutions of barium was Level 0 of the International Dysphagia Diet Standardisation Initiative (IDDSI) [17]. The worst scores were obtained for each scale, as described below. Afterward, 3 g of barium jelly or 5 mL of barium with a commercial xanthan gum-based thickener (IDDSI Level 1) was swallowed. VFSS results were evaluated according to an excerpt on a Japanese scale established by the Japanese Society of Dysphagia Rehabilitation that had been accepted in many Western journals (S1 Table, [9, 10, 12]) and the Dysphagia Outcome and Severity Scale (DOSS) [16, 18, 19]. Using the Japanese scale, the following parameters were assessed: lip closure, bolus formation, bolus transport during the oral phase, pharynx constriction, larynx elevation, bolus stasis at the valleculae and pyriform sinus, and aspiration during the pharyngeal phase. A three-point scale was used to quantify each variable in a VFSS series: 3 (normal), 2 (disturbed), and 1 (severely disturbed). When the Japanese scale was used, the oral phase (3 = severely affected and 9 = normal) and pharyngeal phase (4 = severely affected and 12 = normal) were separately evaluated, and their values were summed to obtain the total score. DOSS (1 = severely affected and 7 = normal) is widely used worldwide but cannot separately evaluate the oral and pharyngeal phases [16]. Each patient was scored independently by one speech language pathologist and one neurologist who were blinded to all clinical details. Both evaluators have more than 10 years of experience in swallowing evaluation. In cases of score discrepancy, an additional neurologist who was also a swallowing expert decided which score was appropriate.

Oral transit time (OTT) was calculated from the time of beginning of backward tongue movement to the time arrival of the bolus head at the ramus of the mandible. Pharyngeal transit time (PTT) was calculated from the time of arrival of the bolus head at the ramus of the mandible until the time that the tail of the bolus passed through the upper esophageal sphincter [4]. OTT and PTT were measured during the second swallow of 5 mL of diluted barium in all patients. OTT and PTT were evaluated by a fixed assessor blinded to clinical data. Institutional normal values of OTT and PTT were obtained from our previous study using six healthy controls (aged 65 ± 14 years) as the standard [12].

### Evaluation of parkinsonism

Parkinsonism was evaluated using the Hoehn–Yahr scale and part III (motor examination) of the United PD Rating Scale (UPDRS-III) according to medical charts. All patients were evaluated before and 32 ± 5 days (mean ± SD) after safinamide treatment. Patients were evaluated during on-time.

### Statistical analyses

The abovementioned scores and times were compared between pre- and post-treatment. Ordinal data, such as total scores, scores during the oral and pharyngeal phases, and DOSS scores were subjected to Wilcoxon signed-rank test [12]. OTT and PTT, which were not distributed normally in our previous studies, served as continuous values [10, 12]; these parameters were also subjected to Wilcoxon signed-rank test. These six measures were set as the primary outcomes for swallowing function on safinamide treatment. P values were adjusted for multiple comparisons using Benjamini and Hochberg’s false discovery rate [20–22] and were calculated using R version R4.2.2 and R commander, designed to add statistical functions frequently used in biostatistics [23]. Critical values were calculated with a false discovery rate of 5%. For exploratory outcomes, all components of the Japanese scale were examined to determine the significant contribution to differences in either oral score or pharyngeal score. Although these components were analyzed using the Wilcoxon signed-rank test, statistically significant levels were not set in this study given that we did not adjust the p values in the exploratory outcomes. For another exploratory outcome, the differences of motor function scores and those of VFSS were analyzed by Spearman correlation coefficient, without p-value adjustment; hence statistically significant levels were not set in this study. The non-parametric effect size (r) was calculated by dividing the absolute standardized statistic (z) of the Wilcoxon signed-rank test by the square root of the pair number (z/√N) [24]. Interpreting the “r” values was as follows: small effect size (r < 0.3), moderate effect size (r = 0.3–0.8), and large effect size (r > 0.8) [24]. Because nonparametric statistics were applied in this study, the Spearman correlation coefficient was used to assess the intra- and inter-rater reliability of the three evaluators for total scores, scores during the oral and pharyngeal phases, and DOSS scores [25]. In addition, inter-rater reliability of the three evaluators was analyzed by weighted Cohen’s kappa coefficient, which was performed using R version R4.2.2 and R commander. We considered a p value of < 0.05 statistically significant during intra- and interrater reliability analyses. P-value adjustment due to multiple comparisons was not necessary in the analyses given that significant relations in any tests were hypothesized for all raters (no inflation of the p value occurred). Statistical analyses were also performed using SPSS software version 22.

## Results

### Improvement in the swallowing function of nine patients with PD

**Fig 1:**
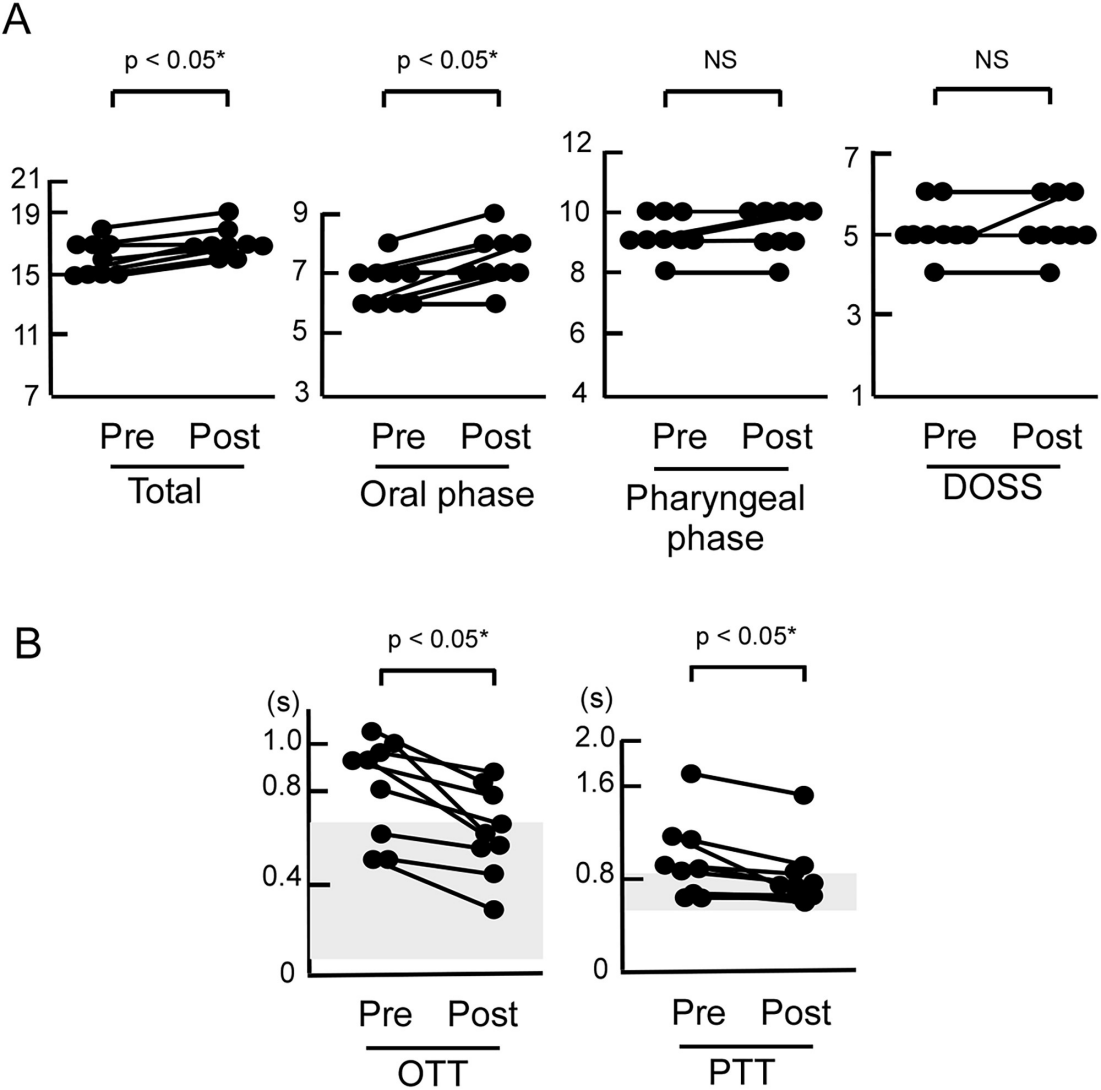
Swallowing function in nine patients with PD before (pre) and 32 ± 5 days after (post) treatment with safinamide. (A) Videofluoroscopic swallowing study (VFSS) showed significant improvements in total score and oral phase score. (p values were calculated using the Wilcoxon signed-rank test; *, significant improvement after adjusting for multiple comparisons using the Benjamini–Hochberg method) Pharyngeal phase score and Dysphagia Outcome and Severity Scale (DOSS) score did not reach statistical significance (NS). (B) VFSS results showed improvement in oral transit time (OTT) and pharyngeal transit time (PTT) after treatment (p values were calculated using the Wilcoxon signed-rank test; *, significant improvement after adjusting for multiple comparisons using the Benjamini–Hochberg method). Shaded regions indicate normal ranges for OTT and PTT.

The oral phase (6.9 ± 0.8 vs. 7.7 ± 0.7, *p* < 0.05, significant improvement after adjusting for multiple comparisons using the Benjamini–Hochberg method) and total scores for the Japanese scale were significantly improved in nine patients with PD after safinamide treatment (16.1 ± 1.2 vs. 17.1 ± 0.9, *p* < 0.05, significant improvement after adjusting for multiple comparisons using the Benjamini–Hochberg method, Fig 1A, Table 1, S2 Table). Among the oral phase components, lip closure was considerably improved (*p* = 0.046, Wilcoxon signed-rank test), although the other components did not reach *p* < 0.05. Pharyngeal score or DOSS did not reach statistical significance. OTT was significantly improved (0.811 ± 0.213 s vs. 0.626 ± 0.209 s, *p* < 0.05, significant improvement after adjusting for multiple comparisons using the Benjamini–Hochberg method, Fig 1B, Table 1, S2 Table), and PTT was mildly but significantly improved after safinamide treatment (0.912 ± 0.335 s vs. 0.820 ± 0.284 s, *p* < 0.05, significant improvement after adjusting for multiple comparisons using the Benjamini–Hochberg method, Fig 1B, Table 1, S2 Table). Effect sizes for all measures were medium or large (Table 2).

**Table 1:** Swallowing measures and motor functions before and after safinamide administration.

	Before	After	Unadjusted p value^#^	Adjusted p value
Oral phase score	6.9 ± 0.8	7.7 ± 0.7	0.020	0.030*
Pharyngeal phase score	9.2 ± 0.7	9.4 ± 0.7	0.157	0.190
Total score	16.1 ± 1.2	17.1 ± 0.9	0.014	0.028*
DOSS	5.1 ± 0.6	5.2 ± 0.7	0.317	0.317
OTT (s)	0.811 ± 0.213	0.626 ± 0.209	0.012	0.028*
PTT (s)	0.912 ± 0.335	0.820 ± 0.284	0.012	0.028*
UPDRS part III	19 ± 3	15 ± 4	nd^§^	nd
Hoehn–Yahr grade	2.8 ± 0.4	2.4 ± 0.5	nd^§^	nd

Values are mean ± SD;

^#^, p values were calculated using Wilcoxon signed-rank test;

*, Significant improvement after adjustment for multiple comparisons using Benjamini–Hochberg method;

^§^, Statistical analyses were not performed.

**Table 2:** Effect sizes of the results of VFSS after safinamide treatment.

	Absolute Z score	r	Effect
Oral phase score	2.333	0.78	Medium
Pharyngeal Phase score	1.414	0.47	Medium
Total score	2.46	0.82	Large
DOSS	1	0.33	Medium
PTT	2.524	0.84	Large
OTT	2.668	0.89	Large

N = 9

### Improvements in motor function as evaluated by the Hoehn–Yahr scale and UPDRS-III

Motor function improved after safinamide treatment in all nine patients as indicated by the UPDRS-III score (20 ± 3 vs. 15 ± 3); however, only three patients showed an improvement in the Hoehn–Yahr scale score. Changes in the UPDRS-III score were not considerably associated with those in the Japanese scale score, DOSS score, OTT, or PTT (S1 Fig).

### Intra- and inter-rater reliability

Intrarater variation analyses revealed that the scores during the oral and pharyngeal phases were significantly associated within each evaluator (*p* < 0.05, Spearman correlation coefficient). Total scores and DOSS scores were significantly associated within each evaluator (*p* < 0.05, Spearman correlation coefficient). Similarly, OTT and PTT were significantly associated within the evaluator (*p* < 0.05, Spearman correlation coefficient). Inter-rater reliability analyses revealed that total scores, scores during the oral and pharyngeal phases, and DOSS scores were significantly associated between evaluators (*p* < 0.05, Spearman correlation coefficient). Inter-rater reliability analyses using weighted Cohen’s kappa coefficient also revealed that total scores, scores during the oral and pharyngeal phases, and DOSS scores were significantly associated between evaluators (*p* < 0.05, S3 Table).

## Discussion

This open-label evaluator-blinded study in nine patients with PD showed that safinamide partly but significantly improved swallowing functions in the current VFSS evaluation. Medicines for PD improve swallowing during the oral phase, which is characterized mainly by voluntary movements [6]. Consistently, our quantitative analysis revealed an improvement during the oral phase, which was also supported by the promoted OTT. The reduced UPDRS-III score implied an improvement in voluntary movements affected by parkinsonism. However, the extent of improvement in swallowing function was not correlated with that of the UPDRS-III score, suggesting that other factors, including impairment of pharyngeal sensation [5] and the reported discrepancy between limb and cranial motor symptoms may prevent the correlation [26]. Safinamide improved PTT, which may reflect smooth bolus transportation during the pharyngeal phase [4]. In the present study, no significant increase in pharyngeal phase scores was observed, suggesting that the continuous values of PTT detect smaller differences than the ordinal values of pharyngeal phase scores. Alternatively, PTT and pharyngeal scale scores are indicators with different aspects, where the latter reflects spatial kinematics and physiological events.

This study provides additional evidence that CDS may be beneficial for swallowing function in PD [27]. Safinamide inhibits MAOB and increases the neuronal dopamine level [27], presumably reducing pulsatile dopaminergic stimulation. The rotigotine transdermal patch improves swallowing when administered as monotherapy and add-on therapy [9, 10]. Transdermal administration may be ideal for CDS. Dopaminergic medicines that provide CDS can prevent or minimize motor fluctuations, including on–off and wearing-off phenomena, in experimental animals [28]. The appearance of off-time during motor fluctuations almost always impairs swallowing [29]. In this study, only one-third of the patients had apparent wearing-off phenomena, which precluded the statistical analyses of motor fluctuations and swallowing functions. Thus, we speculate that safinamide can facilitate CDS in an add-on therapy to improve the swallowing function. Given that larger effect sizes provide strong evidence in favor of therapeutic efficacy, the observed moderate or large effect sizes for all measures might warrant future large-scale studies of safinamide on dysphagia.

The limitations of this study included the small number of mid- to late-stage patients enrolled. In particular, the number of patients with apparent wearing-off phenomena was small. In addition, patients with relatively mild dysphagia were enrolled. Because the small sample size tends to inflate effect sizes, the findings should be interpreted with caution.

In conclusion, a medicine for CDS, safinamide, may be effective in improving swallowing functions in PD. To the best of our knowledge, this is the first study to show that safinamide significantly improves swallowing function, as evaluated using VFSS in patients with PD. Future studies are needed to clarify the effects of safinamide on dysphagia in patients with further advanced-stage PD.

## Supporting information

S1 Table VF scores on the Japanese scale established by the Japanese society of dysphagia rehabilitation. (DOCX)

S2 Table All swallowing measures and motor functions before and after safinamide administration. (DOCX)

S3 Table Coefficient values and p values of weighted Cohen’s kappa coefficient analysis for three evaluators. (DOCX)

S1 Fig The results of Spearman correlation coefficient analyses between motor functions and VSSS findings. No apparent relationships were observed between the difference of UPDRS and those of VFSS results before and after safinamide treatment.
(DOCX)
