# Supplementary material for: The effects of safinamide on dysphagia in Parkinson’s disease
Source: PLoS One. 2023 May 25;18(5):e0286066. doi: 10.1371/journal.pone.0286066 (PMC10212188; doi:10.1371/journal.pone.0286066)
Supplement: S1 Table — (DOCX) [file pone.0286066.s001.docx]

| S1 Table. VF scores on the Japanese scale established by the Japanese Society of Dysphagia Rehabilitation | | | | |
| --- | --- | --- | --- | --- |
|  |  |  |  |  |
|  | **Oral phase (subtotal = 9)** | |  |  |
|  |  | Lip closure | 3・2・1 |  |
|  |  | Bolus formation | 3・2・1 |  |
|  |  | Bolus transportation | 3・2・1 |  |
|  | **Pharyngeal phase (subtotal = 12)** | |  |  |
|  |  | Pharynx constriction | 3・2・1 |  |
|  |  | Larynx elevation | 3・2・1 |  |
|  |  | Bolus stasis at valleculae and pyriform sinus | 3・2・1 |  |
|  |  | Aspiration | 3・2・1 |  |
|  | **Total score (21)** | |  |  |
|  | VF, videofluoroscopy; Parenthesis indicates normal values; 3 = normal; 2 = mildly affected; 3 = severely affected. | | | |
