# Supplementary material for: The effects of safinamide on dysphagia in Parkinson’s disease
Source: PLoS One. 2023 May 25;18(5):e0286066. doi: 10.1371/journal.pone.0286066 (PMC10212188; doi:10.1371/journal.pone.0286066)
Supplement: S2 Table — (DOCX) [file pone.0286066.s002.docx]

| S2 Table. All swallowing measures and motor functions before and after safinamide administration | | | | | | | | | | |  |
| --- | --- | --- | --- | --- | --- | --- | --- | --- | --- | --- | --- |
| Patient# | Age at examination (y) | Sex | Age at onset (y) | Oral score Pre | Oral Score post | Pharyngeal Pre | Pharyngeal Post | Total  pre | Total post | DOSS  pre | DOSS post |
| 1 | 73 | F | 67 | 6 | 7 | 9 | 9 | 15 | 16 | 5 | 5 |
| 2 | 88 | F | 83 | 7 | 8 | 10 | 10 | 17 | 18 | 5 | 5 |
| 3 | 70 | F | 64 | 7 | 7 | 10 | 10 | 17 | 17 | 5 | 5 |
| 4 | 79 | M | 70 | 6 | 7 | 9 | 10 | 15 | 17 | 6 | 6 |
| 5 | 85 | F | 79 | 8 | 9 | 10 | 10 | 18 | 19 | 5 | 5 |
| 6 | 74 | M | 73 | 7 | 7 | 9 | 10 | 16 | 17 | 6 | 6 |
| 7 | 82 | F | 72 | 7 | 8 | 8 | 8 | 15 | 16 | 4 | 4 |
| 8 | 74 | F | 69 | 6 | 8 | 9 | 9 | 15 | 17 | 5 | 6 |
| 9 | 74 | M | 72 | 8 | 8 | 9 | 9 | 17 | 17 | 5 | 5 |
| Pre, Before safinamide administration; Post, After safinamide administration; DOSS, Dysphagia Outcome and Severity Scale; OTT, Oral transit time; PTT, Pharyngeal transit time; H-Y, Hoen-Yahr scale score; UPDRS-III, Part III (motor examination) of the United PD Rating Scale. | | | | | | | | | | | |

| S2 Table. All swallowing measures and motor functions before and after safinamide administration | | | | | | | |  |
| --- | --- | --- | --- | --- | --- | --- | --- | --- |
| Patient#1 | OTT pre (s) | OTT post (s) | PTT pre (s) | PTT post (s) | H-Y pre | H-Y post | UPDRS-III pre | UPDRS-III post |
| 1 | 0.997 | 0.634 | 0.835 | 0.734 | 3 | 2 | 23 | 12 |
| 2 | 0.630 | 0.564 | 1.101 | 0.901 | 3 | 2 | 17 | 12 |
| 3 | 0.809 | 0.666 | 0.633 | 0.632 | 3 | 3 | 20 | 13 |
| 4 | 0.500 | 0.236 | 0.634 | 0.634 | 3 | 3 | 20 | 18 |
| 5 | 0.500 | 0.430 | 0.668 | 0.601 | 3 | 2 | 20 | 14 |
| 6 | 0.966 | 0.873 | 1.702 | 1.532 | 2 | 2 | 14 | 10 |
| 7 | 0.930 | 0.567 | 0.831 | 0.769 | 3 | 3 | 22 | 20 |
| 8 | 1.033 | 0.868 | 0.968 | 0.768 | 2 | 2 | 20 | 17 |
| 9 | 0.937 | 0.798 | 0.840 | 0.810 | 3 | 3 | 24 | 18 |
| Pre, Before safinamide administration; Post, After safinamide administration; DOSS, Dysphagia Outcome and Severity Scale; OTT, Oral transit time; PTT, Pharyngeal transit time; H-Y, Hoen-Yahr scale score; UPDRS-III, Part III (motor examination) of the United PD Rating Scale. | | | | | | | | |
