# Supplementary material for: The effects of safinamide on dysphagia in Parkinson’s disease
Source: PLoS One. 2023 May 25;18(5):e0286066. doi: 10.1371/journal.pone.0286066 (PMC10212188; doi:10.1371/journal.pone.0286066)
Supplement: S3 Table — (DOCX) [file pone.0286066.s003.docx]

| S3 table. Coefficient values and *p* values of weighted Cohen's kappa coefficient analysis for three evaluators | | | |
| --- | --- | --- | --- |
|  |  |  |  |
|  | Evaluator 1 vs evaluator 2 | Evaluator 1 vs evaluator 3 | Evaluator 2 vs evaluator 3 |
| Oral phase score | 0.69, 0.0000045 | 0.92, 0.0000000012 | 0.68, 0.000018 |
| Pharyngeal phase score | 0.54, 0.00031 | 0.51, 0.0015 | 0.529, 0.00050 |
| Total score | 0.46, 0.000061 | 0.53, 0.0000034 | 0.56, 0.000069 |
| DOSS | 0.70, 0.00077 | 0.62, 0.0015 | 0.72, 0.00017 |
| Coefficient value, *p* value |  |  |  |
