# Supplementary material for: The effects of safinamide on dysphagia in Parkinson’s disease
Source: PLoS One. 2023 May 25;18(5):e0286066. doi: 10.1371/journal.pone.0286066 (PMC10212188; doi:10.1371/journal.pone.0286066)
Supplement: S1 Fig — No apparent relationships were observed between the difference of UPDRS and those of VFSS results before and after safinamide treatment. (DOCX) [file pone.0286066.s004.docx]

**Supplemental figure**

**
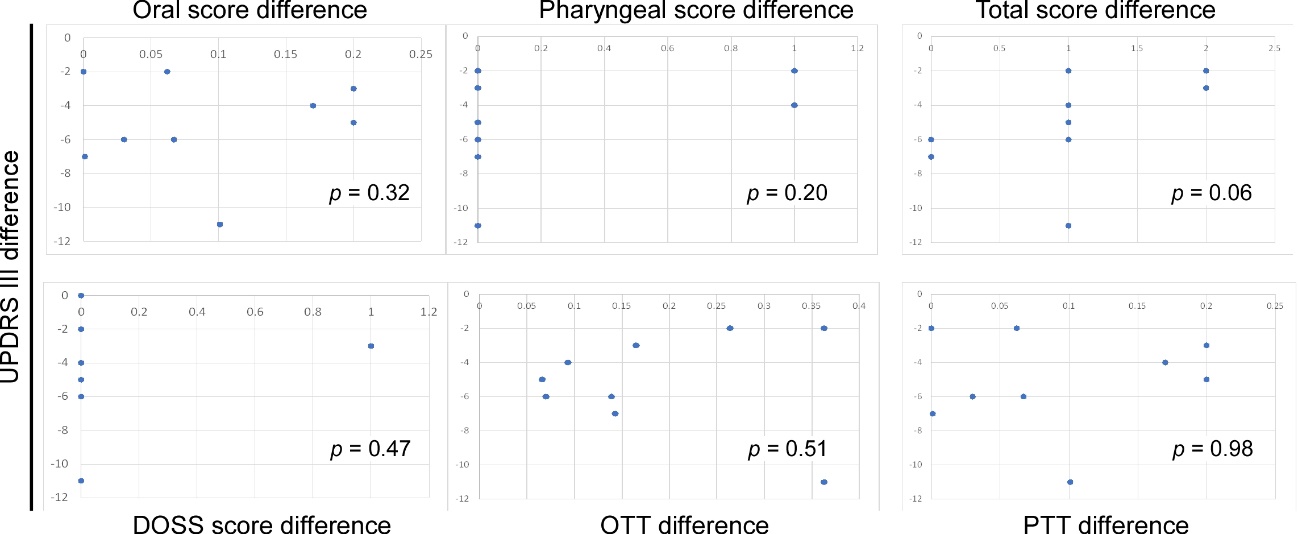
**

S1 Figure. The results of linear regression analyses motor functions and VSSS. No apparent correlation was observed between the difference of UPDRS and VFSS results before and after safinamide treatment.
